# Odors Bias Time Perception in Visual and Auditory Modalities

**DOI:** 10.3389/fpsyg.2016.00535

**Published:** 2016-04-22

**Authors:** Zhenzhu Yue, Tianyu Gao, Lihan Chen, Jiashuang Wu

**Affiliations:** ^1^Department of Psychology, Sun Yat-sen UniversityGuangzhou, China; ^2^Department of Psychology and Beijing Key Laboratory of Behavior and Mental Health, Peking UniversityPeking, China; ^3^Key Laboratory of Machine Perception (Ministry of Education), Peking UniversityBeijing, China

**Keywords:** time perception, odor, visual, auditory, adaptation

## Abstract

Previous studies have shown that emotional states alter our perception of time. However, attention, which is modulated by a number of factors, such as emotional events, also influences time perception. To exclude potential attentional effects associated with emotional events, various types of odors (inducing different levels of emotional arousal) were used to explore whether olfactory events modulated time perception differently in visual and auditory modalities. Participants were shown either a visual dot or heard a continuous tone for 1000 or 4000 ms while they were exposed to odors of jasmine, lavender, or garlic. Participants then reproduced the temporal durations of the preceding visual or auditory stimuli by pressing the spacebar twice. Their reproduced durations were compared to those in the control condition (without odor). The results showed that participants produced significantly longer time intervals in the lavender condition than in the jasmine or garlic conditions. The overall influence of odor on time perception was equivalent for both visual and auditory modalities. The analysis of the interaction effect showed that participants produced longer durations than the actual duration in the short interval condition, but they produced shorter durations in the long interval condition. The effect sizes were larger for the auditory modality than those for the visual modality. Moreover, by comparing performance across the initial and the final blocks of the experiment, we found odor adaptation effects were mainly manifested as longer reproductions for the short time interval later in the adaptation phase, and there was a larger effect size in the auditory modality. In summary, the present results indicate that odors imposed differential impacts on reproduced time durations, and they were constrained by different sensory modalities, valence of the emotional events, and target durations. Biases in time perception could be accounted for by a framework of attentional deployment between the inducers (odors) and emotionally neutral stimuli (visual dots and sound beeps).

## Introduction

Time perception is an important aspect of human life. Although time perception is an important ability for human survival, people often overestimate or underestimate the actual duration of events. Both temporal and non-temporal factors contribute to biases in time estimation. One famous example of temporal factors is from *Vierordt's Law*, which states that judgments of relatively short time intervals are lengthened while the judgments of relatively long time intervals are shortened (Bueti et al., 2008; Block and Gruber, 2014). Non-temporal factors that affect time perception include sensory modality (Goldstone and Lhamon, 1974; Gruber and Block, 2013), emotion states (Droit-Volet and Meck, 2007; Noulhiane et al., 2007; Tipples, 2008; Wittmann and Paulus, 2008; Droit-Volet and Gil, 2009; Gil and Droit-Volet, 2011; Lee et al., 2011), dynamic features of stimuli (Kanai et al., 2006), and directions of motion stimuli (Ono and Kitazawa, 2010). For the modality effect, previous studies have shown that individuals tend to perceive durations as longer in the auditory modality than in the visual modality when the physical durations are less than 1 s (Goldstone and Lhamon, 1974; Wearden et al., 1998). However, the difference between time estimations in the visual and auditory modalities decreased or disappeared when the stimulus duration was longer than 3–5 s (Gruber and Block, 2013; Block and Gruber, 2014). Those differential effects indicate that the illusory bias in time perception is duration-selective.

Among the non-temporal factors, attentional factors and their modulations of the “internal clock” have mostly been exploited to account for the bias in time perception. The traditional internal clock model, which mainly consists of a pacemaker and an accumulator as well as devices of memory and decision components, could explain the processing of time estimation (Gibbon et al., 1984; Buhusi and Meck, 2005). The pacemaker sends out pulses (that is, units of elapsed time) to the accumulator at a particular rate, and the subjectively perceived duration of time is defined by the number of temporal units accumulated over an actual time interval (Schwartz et al., 1986). A close inspection of the internal-clock model suggests that attention and arousal states modulate the accumulation of pulses and cause variations in subjective time estimation (Wittmann and Paulus, 2008). On one hand, increased attentional focus on time perception led to an accumulation of more pulses (Schreuder et al., 2014). On the contrary, when attention was attracted by other task-irrelevant factors, fewer pulses were calculated, and the perceived duration was thus shorter for a given time interval (Droit-Volet and Meck, 2007). On the other hand, increased arousal led to the increased rate of pulses emitted by the pacemaker and induced a greater/faster accumulation of pulses over time. Thus, a given time interval tended to be perceived as longer in a high than in a low arousal condition.

By using emotional faces, Zhang and Zhou (2007) investigated the influence of emotional events on time perception. They found that participants underestimated the duration of angry faces but overestimated the duration of happy faces. Although, arousal states were used to explain their results, their results could also be explained by the distribution of attentional resources between temporal and non-temporal processing. Processing emotional information and estimating time intervals share common attentional resources. When emotional events captured attention, attentional resources allocated for processing time information were reduced. Hence, the perceived subjective time was shorter than the actual duration due to the loss of pacemaker pulses. In the above paradigm, the target stimuli for time estimation coupled emotional information and attentional factors, which made it difficult to tease apart the roles of the different variables (attention vs. emotion) and to exclude the potential confounding variables (such as “emotional states”) induced by the target stimuli themselves.

To overcome this potential confound, an ideal experimental design would be to implement/modulate the arousal states from a third sensory modality while investigating time perception in the target modality. To achieve this, in the current study, we investigated time perception in visual and auditory modalities while manipulating the arousal states in a third modality, namely olfaction, to minimize the emergent properties of emotional information associated with the targets and to examine the other modulating factors beyond arousal states that would affect time perception.

It has been well documented that odors can induce different arousal experiences. For example, odors with positive emotional experience, such as lavender, chamomile, and sandalwood, can decrease anxiety levels (Schwartz et al., 1986; Roberts and Williams, 1992; Moss et al., 2003). In contrast, jasmine and rosemary have been shown to improve alertness and enhance cognitive performance (Kovar et al., 1987; Diego et al., 1998). By using a priming paradigm, Gros et al. (2015) investigated the influence of emotional prime stimuli on the duration estimation of a target. Participants estimated the duration of a pure sound, which was primed by odors or emotional videos. Their results showed that odors consistently activated the arousal system because the measured skin conductance (SC) increased consistently, and no decrease in SC was observed across time. Their results suggest that odors could be well used to investigate the arousal-related mechanism. However, a previous study has explored the effect of odor on time perception (Schreuder et al., 2014), and a time distortion was still observed even though no increase in arousal was indicated by SC or heart rate. In this study, participants were assigned to the rosemary (arousing), peppermint (relaxing), or no odor (control) condition, and they sat either upright (arousing) or lied down (relaxing) during the time perception task. Participants estimated the lengths of time intervals (1.33, 1.58, and 2.17 min) and produced the durations by clicking a mouse button twice to mark the beginning and end of the time periods. Their results showed that the participants produced shorter time intervals in the rosemary odor condition than in the no odor condition, suggesting that odors impacted time perception. However, it should be noted that all time intervals used in this experiment exceeded 1 min, which made the exploration of the timing mechanism illusive when the target time interval was shorter. According to Fraisse (1984), estimating time duration longer than 5 s would mainly exploit a cognitive mechanism and need long-term memory. Therefore, it is reasonable to assume that estimating time durations of less than 5 s might tap into different cognitive resources/processes and hence have different behavioral patterns than comparing time estimations of long durations, as was conducted in Schreuder and colleagues' study (2014). Therefore, we aimed to investigate how odors influenced estimates of time duration of less than 5 s (Poeppel, 2004) and explored different timing mechanisms within 5 s.

For time perception, although some studies have found that there are differences between auditory and visual signals (Goldstone and Lhamon, 1974; Wearden et al., 1998), others have not found modality differences (Bobko et al., 1977). For example, Penney et al. (2000) investigated the effect of stimulus modality on duration classification with a duration bisection task (Allan and Gibbon, 1991). Visual or auditory signals were timed either simultaneously on some trials or alone on other trials. In the training period, participants were presented either short or long anchor durations of signals. Participants made duration judgments (short or long) in the testing period in which there were two anchors and five geometrically spaced intermediate probe durations. They found that modality effect was only observed in blocks containing only a single modality condition, but it was not observed when participants experienced both modalities in the same block. Their results indicated that the temporal precision across sensory modalities is different (Welch and Warren, 1980), and an internal clock runs at a faster rate for auditory than for visual signals. The main purpose of the present study was to investigate to what extent the perception of visual or auditory stimulus duration was influenced by the presence of odors within the time range less than 5 s. To achieve this purpose, two odors of positive affect (jasmine- high arousal; lavender- low arousal) and one odor of negative affect (garlic-high arousal) were used. To increase the accuracy of duration reproductions, a sample time interval was presented first, and then participants produced the same time intervals in the present study. Participants were shown a dot or heard a tone for either 1000 or 4000 ms. After the stimulus, participants estimated the stimulus duration by pressing the space bar twice to demarcate the beginning and end of a produced time duration. We hypothesized that the arousal level induced by the odors would affect the perceived duration in both the visual and auditory modalities. Individuals perceived a given time interval as longer in the high arousal condition than in the low arousal condition (Tremblay and Fortin, 2003) because the arousal states quicken the accumulation of pulses. Moreover, the perceived duration was also influenced by the attention mechanism when attentional resources were not depleted and could be directed to the targets because olfactory stimuli were presented simultaneously.

As in the other sensory modalities, exposure to the odors for a long time period would lead to sensory adaptation and change the subjective sensitivities to the odors. If any emotional states were triggered by the odors, they would influence the time perception for target events as a function of the passage of time. Hence, we compared performances of the initial and the final parts in the experiment, which were separated from each other by approximately by approximately 7–10 min, to show the potential bias in time across the different adaptation phases.

## Materials and methods

### Participants

One hundred undergraduate students (29 males, 71 females) from Sun Yat-sen University participated in this study. They were 17–24 years old (Mean age = 19.7, SD = 1.40). Data from two participants were excluded due to exceeding three standard deviations of the average. Therefore, the final analysis consisted of data from 98 participants, including 23 participants (10 males) in the jasmine condition, 23 participants (8 males) in the lavender condition, 24 participants (6 males) in the garlic condition, and 28 participants (5 males) in the no odor condition.

All participants were right handed except for one. Participants self-reported normal olfaction, audition and normal or corrected-to-normal vision. They were paid 10 Chinese yuan for taking part in the study. The study was conducted in accordance with the guidelines in the Declaration of Helsinki (2000) and was approved by the Ethics Committee of Department of Psychology, Sun Yat-sen University. All participants gave their written informed consent before taking part in the study.

### Stimuli

#### Olfactory stimuli

Three odors (garlic, jasmine, and lavender) were used. The garlic odor was picked from a solution prepared by dissolving 525 g of garlic odorizor powder into 300 ml of water. The jasmine and lavender odors were made from 300 ml of liquid air fresheners with the respective fragrance. No negative low arousal odor was used because we were focusing on the categories of “positive” and “negative” odors. Moreover, most negative odors are highly arousing, and it is not convenient to modulate the level of arousal state with negative odors.

To avoid the mixing arousal induced by different odors, only one of the three odors was randomly assigned to each participant. We soaked two cotton pads (6 × 5 cm^2^) in 5 ml of an odor liquid for 20 min. Before the experiment, the experimenter brought the cotton pads into the room and fixed them under the desk. We occluded the cotton pads such that participants only smelled them but could not see them. After the cotton pads had been placed in the room for 30 min, participants entered the room (1.2 × 1.7 m^2^) to start the experiment.

#### Visual and auditory stimuli

The visual stimulus was a white dot (of visual angle 5.27° × 5.27°, Luminance 10.4 cd/m^2^), which was presented on a 17-inch monitor (Refresh rate 60 Hz) and controlled by E-prime (http://www.pstnet.com/eprime.cfm). The auditory stimulus was a pure tone (500 Hz, 70 dB) presented via headphones (EDIFIER H850) to both ears.

### Procedures

Participants sat in front of a monitor in a room, which was dimly lit and windowless. The viewing distance was 60 cm. Participants did not receive any information about the odors before the experiment.

**Figure 1:**
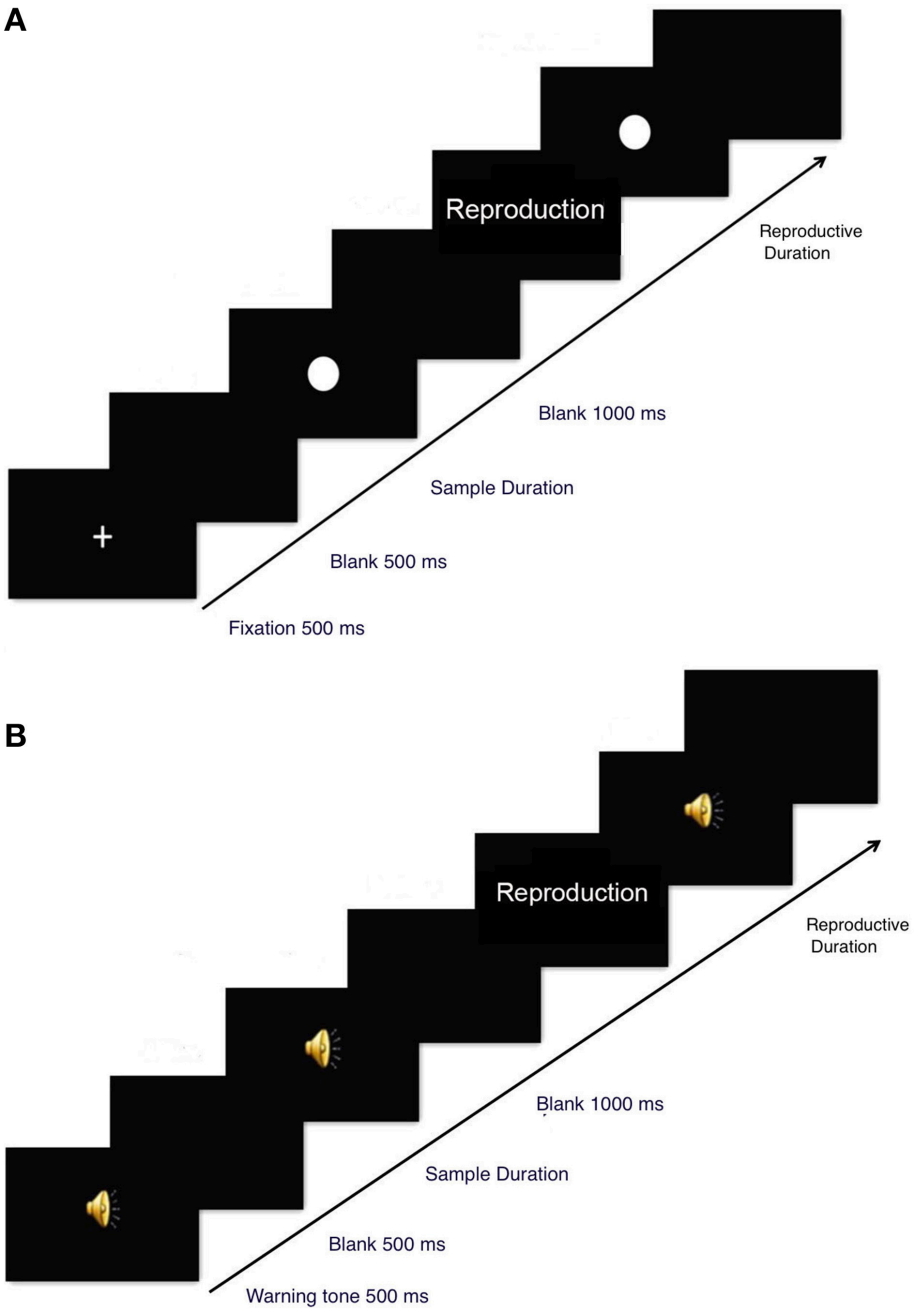
**Stimuli and experimental setup in the visual (A) and auditory (B) conditions**.

In the visual condition, a fixation cross (of visual angle 5.27° × 5.27°) was presented in the center of the monitor for 500 ms followed by a 500 ms blank (see Figure 1A). Next, a white dot was presented in the center of the screen for an average of 1000 ms (randomly selected from 800, 900, 1000, 1100, or 1200 ms) or for an average of 4000 ms (randomly selected from 3800, 3900, 4000, 4100, or 4200 ms). Each duration was presented six times in each condition. Then, the screen turned black and the participant waited for 1000 ms before (s)he reproduced the presentation duration of the white dot. Participants could reproduce the time interval after the word “reproduction” was present on the screen, and the word was kept on the screen until the produced duration was finished. Specifically, when making a response, a participant first pressed the spacebar once, and then a white dot appeared on the screen. (S)he waited for an equivalent length of time that (s)he believed the original visual stimulus duration to be and then pressed the spacebar again to end the trial. The screen then turned black for 1000 ms before a new trial began.

In the auditory condition, a cueing sound (2000 Hz, 500 ms) was delivered via a set of headphones, which was followed by a 500 ms blank (see Figure 1B). Next, a pure tone (500 Hz, 70 dB) was presented for an average of 1000 ms (randomly selected from 800, 900, 1000, 1100, or 1200 ms) or 4000 ms (randomly selected from 3800, 3900, 4000, 4100, or 4200 ms). Participants then waited quietly for 1000 ms and reproduced the duration of the auditory stimulus by pressing the spacebar twice (as in the procedure in visual condition). When participants first pressed the spacebar, a pure tone initiated and lasted until participants pressed the space bar again.

Each participant completed two blocks with visual stimuli and two blocks with auditory stimuli, each consisting of 30 trials. Half of the participants started with the visual task, and the other half started with the auditory task (block orders were counterbalanced between subjects). For each condition, at least 10 practice trials were completed prior to the start of the formal experiment. Participants took a break after the completion of each block.

After the time reproduction task, participants answered the following survey of four questions in the same room: (1) Did you notice the odor in the room? (2) Please specify the type of odor in the room: jasmine, lavender, or garlic. (3) When you smell this odor, please rate your emotional experience on a scale from −4(extremely unpleasant) to 4(extremely pleasant). (4) When you smell this odor, please rate your emotional experience on a scale from −4(extremely calm) to 4(extremely aroused). After a participant answered the questions and left the room, we discarded the cotton pads and ventilated the room for 20 min.

### Data analyses

For the odors used in the present study, participants rated each odor according to its valence and arousal. The olfactory discrimination and evaluation were analyzed with one-way analyses of variance (ANOVA).

For the time reproduction task, the dependent variable was the difference between the estimated duration and the actual duration. A positive value meant longer reproductions of the time interval than the actual duration, and a negative value meant shorter reproductions of the time interval than the actual duration. A ratio score was also calculated with the following formula: [T corrected score = (T estimated − T standard) / T standard] (Brown, 1985). We then performed a 4 (odor type: high-arousal positive odor: jasmine, low- arousal positive odor: lavender, high-arousal negative odor: garlic, and no odor) × 2 (modality: visual vs. auditory) × 2 (interval: short vs. long) ANOVA. The odor type was a between-subjects variable, while the other two were within-subjects variables.

To measure the effect of the adaption to odors, the performance in the time reproduction task was compared between the initial and the final block for each modality. A four-way repeated measures ANOVA was conducted, with another within-subjects variable, adaptation phase (initial block vs. final block), in addition to the above three factors: odor type, modality and interval.

## Results

### Emotion induction by each odor

Participants in the three odor conditions (not including those in the no-odor condition) rated the valance and arousal levels of each odor. All participants noticed the odor in the room and identified the odors correctly.

**Figure 2:**
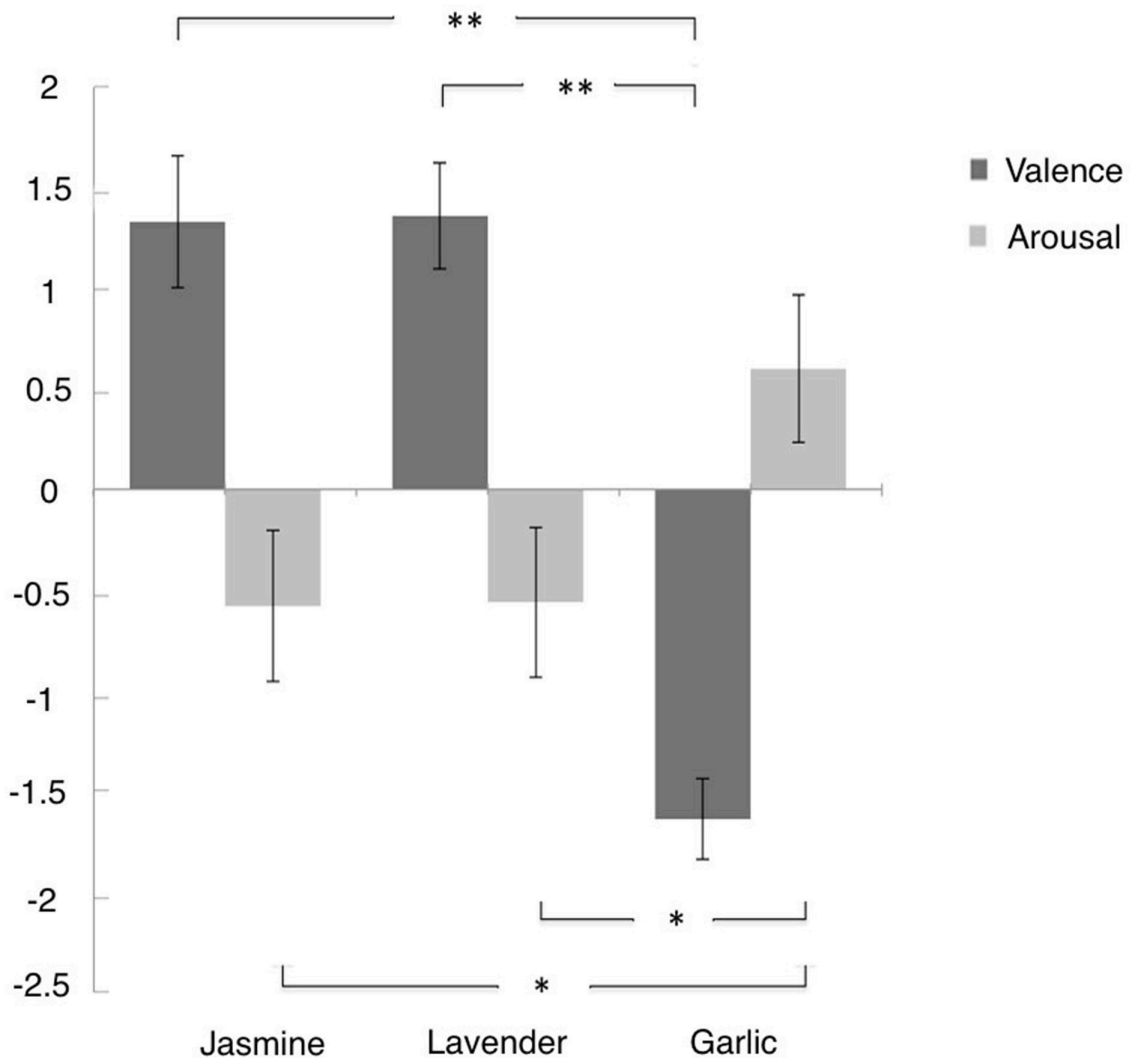
**The mean value of self-reported valence and arousal of three odors**. Error bars indicate the standard deviation. Participants rated their emotional experience on a scale from −4 to 4. ^*^p < 0.05, ^**^p < 0.01.

The valence and arousal scores were summarized in Figure 2. One-way ANOVA revealed a main effect of odor by valance, *F*_(2, 67)_ = 41.4, *p* < 0.0001, η^2^ = 0.553, and a marginal main effect of odor on arousal, *F*_(2, 67)_ = 3.07, *p* = 0.053, η^2^ = 0.084. For the emotional valence, further *t*-tests showed that the pleasantness of the jasmine odor (*M* = 1.30, *SE* = 0.34) and lavender odor (*M* = 1.43, *SE* = 0.27) were significantly greater than garlic odor (*M* = −1.66, *SE* = 0.21), *t*_(45)_ = 7.53, *p* < 0.01, Cohen's *d* = 2.181, and *t*_(45)_ = 9.3, *p* < 0.01, *d* = 2.649, respectively. For the emotional arousal, the arousal of the garlic odor (*M* = 0.54, *SE* = 0.38) was significantly greater than the arousal of the jasmine odor (*M* = −0.65, *SE* = 0.39), *t*_(45)_ = 2.2, *p* < 0.05, *d* = 0.638, and the lavender odor (*M* = −0.61, *SE* = 0.40), *t*_(45)_ = 2.08, *p* < 0.05, *d* = 0.609. There was no difference between the jasmine and lavender odors for the emotional valence [*t*_(44)_ = −0.302, *p* = 0.76, *d* = 0.089] or the emotional arousal [*t*_(44)_ = −0.078, *p* = 0.94, *d* = 0.021].

### Time reproduction task

The outlier data of the participants, i.e., the reaction times exceeding three standard deviations (less than 5 %) in each experimental condition, were removed. Table 1 shows the mean differences between the reproduced time intervals and actual time intervals in which the actual duration was subtracted from the reproduced duration. To control for the initial bias for the baseline intervals (short vs. long), the following ratio score was also adopted: T corrected score = (T estimated – T standard)/T standard.

**Table 1:** **The means (ms) and standard errors (se) of the over- or under-estimation of time intervals (the differences between reproductive time intervals and real time intervals) in all experimental conditions. The ratio scores were also calculated and shown in the table**.

**Odor**	**Short interval**		**Long interval**	
	**Auditory**	**Visual**	**Auditory**	**Visual**
**MEAN REPRODUCTION INTERVAL (ms)**				
Jasmine	183.7 (51.9)	35.5 (49.35)	−261.3 (67.33)	−243.3 (70.84)
Lavender	314.2 (51.9)	175.3 (49.35)	−125.1 (67.33)	−4.4 (70.84)
Garlic	134.7 (50.88)	96.4 (48.31)	−232.0 (65.91)	−233.1 (69.35)
No odor	200.8 (47.11)	154.0 (44.72)	−210.4 (61.02)	−121.6 (64.21)
**T CORRECTED SCORE** = **(MEAN REPRODUCTION INTERVAL** − **STANDARD INTERVAL)/STANDARD INTERVAL**				
Jasmine	0.185 (0.049)	0.025 (0.047)	−0.063 (0.016)	−0.054 (0.016)
Lavender	0.267 (0.049)	0.124 (0.047)	−0.029 (0.016)	−0.007 (0.016)
Garlic	0.18 (0.044)	0.143 (0.042)	−0.048 (0.014)	−0.018 (0.014)
No odor	0.108 (0.048)	0.059 (0.046)	−0.051 (0.015)	−0.046 (0.016)

A three-way ANOVA (odor × interval × modality) revealed a significant main effect of odor, *F*_(3, 94)_ = 2.92, *p* < 0.05, η^2^ = 0.085. Further *t*-tests showed that the time estimation bias in the lavender condition (*M* = 92.2 ms, *SE* = 44.0) was significantly greater than that in the jasmine condition (*M* = −71.36 ms, *SE* = 44.0), *t*_(44)_ = −2.84, *p* < 0.01, *d* = 0.775 and greater than that in the garlic condition (*M* = −58.5 ms *SE* = 43.0,), *t*_(45)_ = 2.5, *p* < 0.05, *d* = 0.715. No significant differences between the no odor condition and the three odor conditions were found, and all *p'*s were greater than 0.8. In addition, the main effect of the interval was significant, *F*_(1, 94)_ = 120.75, *p* < 0.01, η^2^ = 0.562. Participants tended to reproduce longer durations than the actual durations for short time intervals (*M* = 161.83 ms, *SE* = 21.76) and reproduce shorter durations for long time intervals (*M* = −177.81 ms, *SE* = 30.32) in all odor conditions, thus resembling *Vierordt's Law*. The main effect of modality did not reach significance, *F*_(1, 94)_ = 0.68, *p* = 0.41.

**Figure 3:**
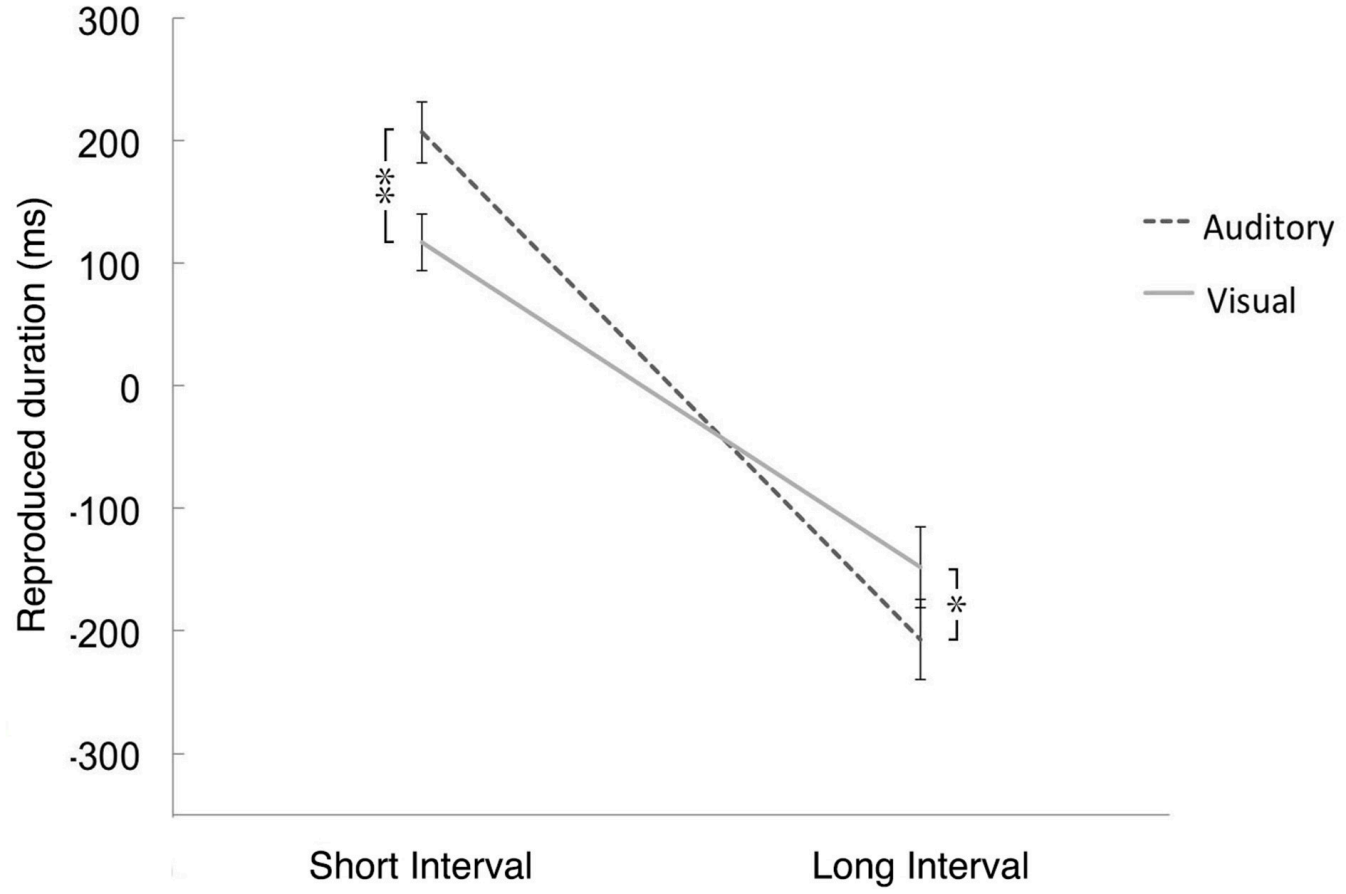
**The mean value of differences between the reproductive time intervals and the actual time intervals in which the actual duration was subtracted for the reproductive duration**. Positive value means longer reproduction of time intervals than the actual duration, while negative value means shorter reproduction of time intervals than the actual duration. Error bars indicate the standard deviation. ^*^p < 0.05, ^**^p < 0.01.

The interaction between the interval and modality was significant, *F*_(1, 94)_ = 22.683, *p* < 0.01, η^2^ = 0.194 (see Figure 3). Further *t*-tests showed that for the short interval condition, the reproductions in the auditory modality (*M* = 207.23 ms, *SE* = 25.62) were significantly longer than those in the visual modality (*M* = 117.08 ms, *SE* = 24.14), *t*_(97)_ = 3.884, *p* < 0.001, *d* = 0.365. By contrast, for the long interval condition, the shorter reproductions of auditory time intervals (*M* = −207.63 ms, *SE* = 32.49) was significantly greater than that of visual time intervals (*M* = −147.91 ms, *SE* = 35.21), *t*_(97)_ = −2.069, *p* < 0.05, *d* = 0.178. The difference between the visual and auditory modalities (*M* = 90.15 ms, *SE* = 23.21) for longer time intervals did not differ significantly from the difference between modalities for shorter intervals (*M* = 59.72 ms, *SE* = 28.87), *t*_(97)_ = 0.738, *p* > 0.05, *d* = 0.117. The interaction between odor, interval and modality did not reach significance, *F*_(3, 94)_ = 2.15, *p* = 0.09.

The analysis of variance (ANOVA) of the ratio scores showed a main effect of interval, *F*_(1, 94)_ = 90.832, *p* < 0.001, η^2^ = 0.491, and a significant main effect of modality, *F*_(1, 94)_ = 10.593, *p* < 0.01, η^2^ = 0.101. The interaction between interval and modality was significant, *F*_(1, 94)_ = 25.045, *p* < 0.001, η^2^ = 0.21 (see Table 1, it has similar trend as in Figure 3). Further tests revealed that for the short interval condition, the ratio score for longer reproductions of the auditory time interval (*M* = 0.185, *SE* = 0.024) was significantly larger than that of the visual time interval (*M* = 0.088, *SE* = 0.023), *t*_(97)_ = 4.05, *p* < 0.0001, *d* = 0.417. By contrast, for the long interval condition, the ratio score for shorter reproductions of the auditory time interval (*M* = −0.048, *SE* = 0.008) was significantly larger than that of the visual time interval (*M* = −0.031, *SE* = 0.008), *t*_(97)_ = −2.727, *p* < 0.01, *d* = 0.215. Moreover, the difference between the visual and auditory modalities for longer time intervals (*M* = 0.097, *SE* = 0.006) was significantly larger than the difference between the two modalities for shorter intervals (*M* = 0.017, *SE* = 0.023), *t*_(97)_ = 4.831, *p* < 0.001, *d* = 0.169. However, the main effect of odor, *F*_(3, 94)_ = 1.965, *p* = 0.125, η^2^ = 0.059, and the interaction between odor, interval and modality did not reach significance, *F*_(3, 94)_ = 1.795, *p* = 0.153, η^2^ = 0.054.

### The adaptation of odors

The effect of odor adaptation on time perception was analyzed by comparing the performance of trials in the initial (the first block) and final (the fourth block) parts. The final experimental block started approximately 7 min after the end of the initial experimental block. The mean differences between the reproduced duration and the actual duration were calculated in each experimental condition. A four-way mixed ANOVA was conducted with the between-subjects factor of odor (jasmine, lavender, garlic, vs. no odor), the within-subjects factors of interval (short vs. long), modality (visual vs. auditory), and the adaptation phase (initial vs. final block). A significant main effect of odor was observed, *F*_(3, 94)_ = 3.341, *p* < 0.001, η^2^ = 0.096, which was in accordance with our earlier results. The main effect of interval was significant, *F*_(1, 94)_ = 41.965, *p* < 0.01, η^2^ = 0.309, which suggested that participants produced longer durations than the actual durations for the short time intervals (*M* = 177.6 ms, *SE* = 23.50) and produced shorter durations for the long time intervals (*M* = −104.77 ms, *SE* = 44.34). The main effect of adaptation phase was significant, *F*_(1, 94)_ = 7.24, *p* < 0.01, η^2^ = 0.072, which suggested that participants reproduced longer time durations than the actual duration in the final block (*M* = 84.2 ms, *SE* = 40.58) than in the initial block (*M* = −11.2 ms, *SE* = 23.45).

**Figure 4:**
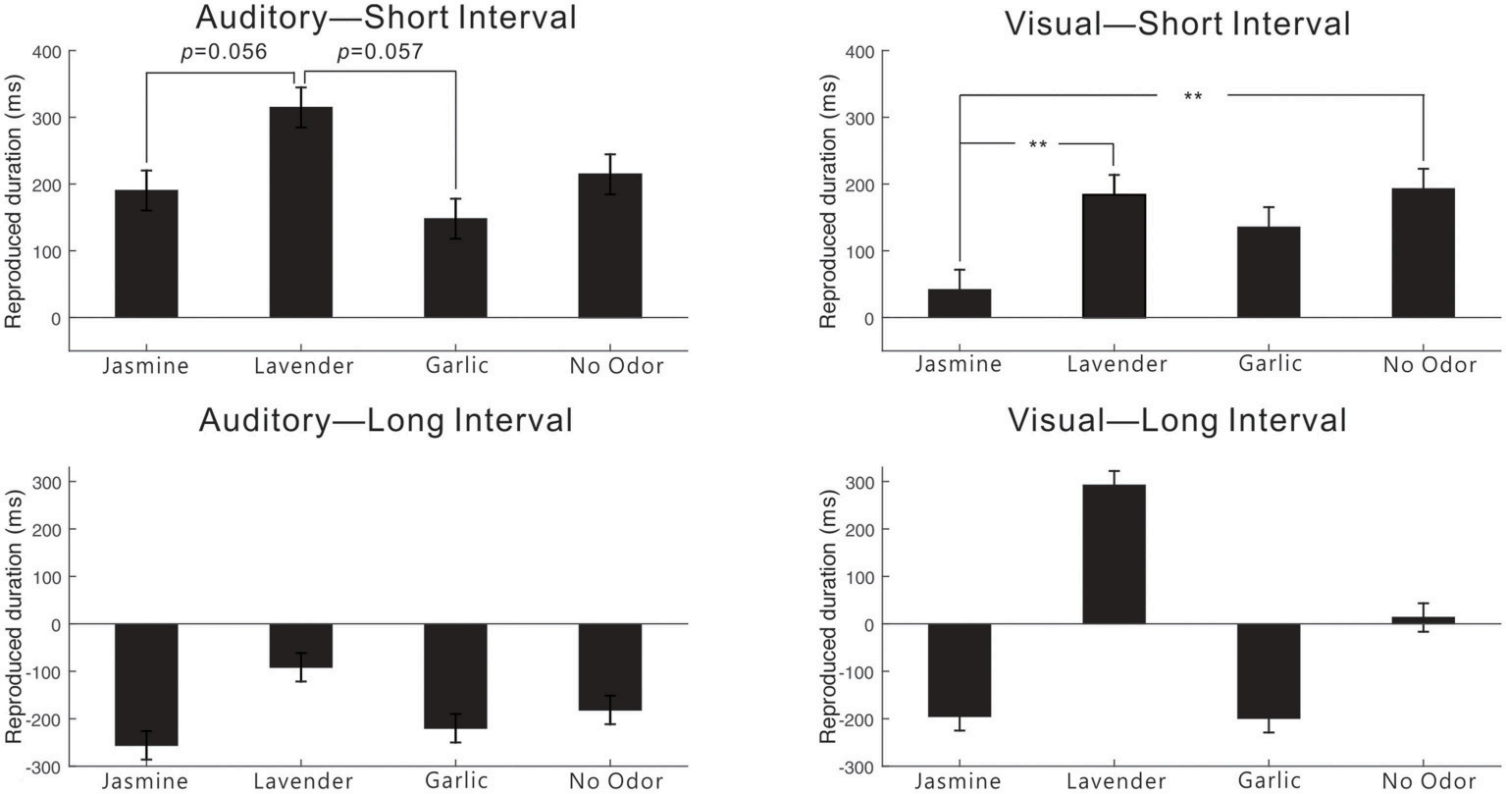
**The mean value of differences between the reproductive time intervals and the actual time intervals for both visual and auditory modalities during the experiment in which the actual duration was subtracted from the estimated duration**. A positive value means a longer reproduction of time intervals than the actual duration, while a negative value means a shorter reproduction of time intervals than the actual duration. Error bars indicate the standard deviation. ^**^p < 0.01.

A significant interaction between modality and interval was observed, *F*_(1, 94)_ = 14.509, *p* < 0.001, η^2^ = 0.134 (see Figure 4). Further *t*-tests showed that in the short time interval condition, the magnitude of the longer reproduction of the auditory time interval (*M* = 216.87 ms, *SE* = 26.53) than the actual time interval was significantly larger than that of the visual time interval (*M* = 138.38 ms, *SE* = 25.61), *t*_(97)_ = 3.25, *p* = 0.002, *d* = 0.304. In contrast, in the long time interval trials, the magnitude of the shorter reproduction of the auditory time interval (*M* = −187.20 ms, *SE* = 34.00) than the actual time interval was significantly larger than that of the visual time interval (*M* = −22.12 ms, *SE* = 69.88), *t*_(97)_ = −2. 532, *p* < 0.05, *d* = 0.303, which was consistent with our earlier results. There is a trend toward significance for the interaction between modality, adaptation phase and odor, *F*_(3, 94)_ = 2.282, *p* = 0.084, η^2^ = 0.068. Further, analyses revealed a significant main effect of adaptation for the no odor condition only, *F*_(1, 27)_ = 4.361, *p* < 0.05, η^2^ = 0.139, indicating that the longer reproduction of time intervals was larger in the final block (*M* = 110.162 ms, *SE* = 74.34) than in the initial block (*M* = 9.479 ms, *SE* = 43.24).

Importantly, there is a trend toward significance for the interaction between modality, interval and odor, *F*_(3, 94)_ = 2.339, *p* = 0.078, η^2^ = 0.069 (see Figure 4). For the short interval condition, further analyses showed a significant main effect of modality, *F*_(1, 94)_ = 12.043, *p* < 0.01, η^2^ = 0.114, and a trend toward significance for the interaction between modality and odor, *F*_(3, 94)_ = 2.476, *p* = 0.066, η^2^ = 0.073. Further, *t*-tests showed that for the auditory modality, the reproduction of the time interval in the lavender condition (*M* = 314.73 ms, *SE* = 54.59) was nearly significantly longer than that in the jasmine (*M* = 190.28 ms, *SE* = 54.59), *t*_(44)_ = −1.959, *p* = 0.056, *d* = 0.475, and garlic conditions (147.95 ms, *SE* = 53.44), *t*_(45)_ = 1.951, *p* = 0.057, *d* = 0.637. For the visual modality, the longer reproduction of the time interval than the actual duration in the jasmine condition (*M* = 41.66 ms, *SE* = 52.69) was significantly smaller than that in the lavender condition (*M* = 183.68 ms, *SE* = 52.69), *t*_(44)_ = −2.245, *p* = 0.03, *d* = 0.562, and no odor condition (*M* = 192.84 ms, *SE* = 47.75), *t*_(49)_ = −2.145, *p* = 0.037, *d* = 0.598. In contrast, for the long interval condition, only a significant main effect of odor, *F*_(3, 94)_ = 2.748, *p* = 0.047, η^2^ = 0.081, and a significant main effect of modality, *F*_(1, 94)_ = 6.469, *p* = 0.013, η^2^ = 0.064, were observed.

## Discussion

The primary goal of the present study was to investigate the influence of different odors on time perception in both visual and auditory modalities. Moreover, we investigated whether the adaptation of odors affected perceived time of different ranges (short vs. long) and the effect sizes across different adaptation phases. In the current study, we used odor stimuli, and the target stimuli (visual dots and sound beeps) were relatively emotionally neutral. Such an olfactory stimulus may be especially suitable for exploring the emotional response by itself because few attentional factors were involved (Gros et al., 2015). Hence, the confounding of attentional alertness induced by the stimuli themselves was minimized. We believe that attentional resources/engagements play an important role in time perception. The current study provides a good avenue to measure the attentional effect because the attentional and emotional factors (including the dimensions of valence and arousal) were separated from the inducers (odors) and the stimuli, thus making the investigations of the roles of attentional deployment and emotional states technically sound. Moreover, our results supported that the emotion induced from one sensory modality influenced time perception in another modality, indicating that there was crossmodal duration modulation (Shi et al., 2012).

Our results revealed a longer reproduction of time intervals than the actual time durations in the lavender condition as well as a shorter reproduction of time intervals in the jasmine and garlic conditions. These results could not be simply explained by arousal. According to the internal clock model (Gibbon et al., 1984), a high level of arousal would accelerate the rate of the pacemaker, increase the pulse of the accumulator, and lead to perceiving the duration as longer than it actually was. However, the present results contradict this prediction. This finding was because in previous studies (Tamm et al., 2014), perceived negative stimuli or threats (such as angry faces) led to the distribution of attentional resources between the tasks of time perception and emotion processing, which impaired time estimation for target events. The seemingly contradictory results indicate that there might be other factors/mechanisms that modulate the bias in time perception. One possible reason is that the valence, rather than the arousal level of the stimuli, may play a major role in modulating time perception across the visual and auditory modalities when the inducers (odors) and target stimuli are separated. As we observed, lavender and jasmine were associated with a “positive” valence, while garlic was associated with a “negative” valence. The positive valence triggered more pulses, which led to a greater overestimation of the produced duration compared to the negative valence. This possibility is potentially weak because we found the opposite patterns with respective to the lavender and jasmine conditions (they had similar ratings for valence and arousal). Alternatively, the high arousal state in the garlic condition attracted attentional resources, which made the neutral stimuli comparatively less attended and decreased the reproduced duration (Tse et al., 2004). In the lavender condition, because the arousal level was low, more attention was directed to the neutral stimuli, and the perceived time intervals were longer. Even with the above arguments, one might consider that the special case of “jasmine” would not support the “attentional” accounts. We reserved the possibility that another dimension, such as personal preference (such as that for “jasmine”), would also attract the attentional resources for processing the time information of target stimuli.

Alternatively, one may also argue for a generally flexible framework for time estimation when different mechanisms, such as attention, arousal (valence), and other modulatory factors, come into play together. An attention mechanism may control the switch/gate, while an arousal mechanism may affect the rate of the pacemaker (Lake, 2016). Each mechanism may compete for general resources to play an important role in the process of time reproduction. For example, previous results have shown that arousal is not the only main mechanism for time distortion because both arousal dependent time distortion (Droit-Volet et al., 2010) and arousal-independent time estimation (Schreuder et al., 2014) were reported. Moreover, attentional deployment might also act on the process of the pacemaker. Specifically, the distortion of time perception may increase according to whether attention is focused on time or on the signals. Furthermore, other factors, such as the gender of the participants (Grondin et al., 2015), anxiety (Bar-Haim et al., 2010), etc. could also modulate the effect size of emotional time distortions. Different mechanisms might counteract each other or neutralize the overall effects. Therefore, in the present study, the fact that we did not observe significant differences between each of the three odor conditions and the no-odor condition might be attributed to this consideration.

The attentional deployment in time perception was also supported by the evidence from the time course of the odor adaptation. In the present study, we found even longer reproductions of time intervals than the actual duration in the final block compared to the initial block of the experiment. An explanation of this finding is that with the passage of time, observers overcame through the influences of the odors, and the attentional resources were re-engaged to the target/neural stimuli (visual dots and auditory beeps). Therefore, we observed longer reproductions of the duration in the final block compared with the reproduced durations in the initial block. Thus, our results support that the function of the attentional mechanism varies over time because attention may be captured by emotional stimuli (Shi et al., 2012) or reduced after the repeated presentation of the emotional stimuli (Gros et al., 2015). It should be acknowledged that there are various adaption processes in the olfactory as well as in the limbic and cognitive systems during long-term smell exposure. Different odors and different dimensional properties of odors also have different time courses of adaptation. The short-spaced interval between the first and the final blocks in the present study could partly reduce the mixing effect of these factors. Nevertheless, further studies may investigate how the adaptation of odors affects time perception.

The attentional mechanism, however, is constrained by the actual length of the target duration. For the effects of odors as well as for their adaptation effect, we found unanimously that the effect sizes were larger in the “short” interval condition than in the “long” interval condition. Moreover, odor adaptation (i.e., the arousing states) influenced the perceived duration differently for the visual and auditory modalities for the short duration (1000 ms) but not for the long duration (4000 ms). As stated in the literature, the two ranges of time intervals (1000 and 4000 ms) may be different with respect to their underlying mechanisms (Poeppel, 1997). For example, the timing of a 1000 ms interval can be considered to be a relatively perceptual process, whereas the timing of a 4000 ms duration may involve higher cognitive functions and is usually referred to as time estimation (Fraisse, 1984; Poeppel, 1997). Moreover, for the short time duration of less than 1 s, the modality effect (i.e., the differences between the visual and auditory modalities) was easily observed. For the auditory signals, the rate of the internal clock was faster than that for the visual signals, thereby inducing longer time perception in the auditory modality.

Although, the present study shed light on multiple mechanisms of emotional time perception, it is important to note that there are some limitations in our study. First, no low negative arousal odor was used in the present study, which made it impossible to interpret the ANOVA results for valence or arousal effects. Because most “negative” odors show high arousal patterns, we did not obtain satisfactory samples for the current study. However, the different and critical arousals and valences are present in the three odors used. To understand the different effect of valences or arousal effects of emotional stimuli, further studies should be conducted in the future. Second, as we noted earlier, for the subjective rating, no difference was found between the jasmine and lavender odors according to the self-report. In the future, to further examine the effect of arousal levels on time perception, the recordings of participants' heart rate, skin conductance (Gros et al., 2015), blood flow and the other physiological indexes may be used to capture the objective evaluation of the “emotional” stimuli. Moreover, we hypothesized that there was a correspondence between the modulating effect of the attentional factor of the inducer and the target stimuli but the exact coupling of the two items requires further study. Finally, we did not find the significant difference between each of three odor conditions and the neutral condition, which might be due to the counteracting effect of the different mechanisms underlying emotional time perception. Further studies in which the emotional stimuli are presented only in an encoding phase and in which the reproduction phase is always neutral should be used (Noulhiane et al., 2007).

In sum, our results show that the perception of time duration is influenced by the presence of inducers (odors). Participants reproduced longer time intervals than the actual durations when exposed to the smell of lavender, and they reproduced shorter time intervals when exposed to the smells of jasmine and garlic. Our results indicated that a mixed mechanism, especially attentional deployment between the inducers (odors) and target stimuli, could largely account for the timing bias across different sensory modalities as well as the timing course of those biases. Those biases, however, were dependent on different target durations and showed that the processing of short and long intervals might use different mechanisms.

## Author contributions

ZY and TG designed the research; TG performed the research; TG, JW, and ZY analyzed the data; ZY and LC wrote the manuscript. All authors commented on and edited the manuscript. All authors approved it for publication.

### Conflict of interest statement

The authors declare that the research was conducted in the absence of any commercial or financial relationships that could be construed as a potential conflict of interest. The handling Editor declared a current collaboration with one of the authors [LC] and states that the process nevertheless met the standards of a fair and objective review.
